# Supply organ development of young broilers in response to increased carbohydrates and amino acids in the starter period

**DOI:** 10.1016/j.psj.2024.104092

**Published:** 2024-07-18

**Authors:** J.J.E. Diehl, E. van Eerden, M. Duijster, R.P. Kwakkel

**Affiliations:** ⁎ Animal Nutrition Group, Department of Animal Sciences, Wageningen University, 6700AH Wageningen, The Netherlands; † Schothorst Feed Research, 8200AM Lelystad, The Netherlands; ‡ Global Nutrition Department, De Heus Animal Nutrition B.V., 6710BJ Ede, The Netherlands

**Keywords:** broiler, supply organ, carbohydrate, development

## Abstract

The growth of broiler chickens is marked by high fluctuations, varying nutrient requirement, early growth is characterized by high allometric growth rates of supply organs, which if underdeveloped, can impede nutrient efficiency and growth of demand organs like muscle and skeleton. This study aimed to investigate the impact of carbohydrate- and amino-acid-rich diets on the development of supply organs in broiler chickens. Four dietary treatments were used in a 2 × 2 factorial arrangement of treatments with apparent metabolizable energy (**AME**) at 2 levels (low: 2,750 kcal/kg and high: 3,050 kcal/kg) and standardized ileal digestible (**SID**) lysine at 2 levels (low: 1.0% and high: 1.2%) in the starter diets. Feed intake (**FI**) and BW gain were measured weekly; dissections were conducted at d 4 and d 11 to determine supply organ weights. Allometric growth of the liver was higher (*P* < 0.001) in the high AME and low lysine group compared to the other groups. For the pancreas, the highest (*P* < 0.001) allometric growth rate was in the high lysine groups. The small intestines responded differently; the duodenum had the highest (*P* < 0.001) allometric growth rate in the high AME groups and the jejunum in the low lysine groups, whereas the ileum showed an effect of diet density. For performance, high AME from carbohydrates, via maize starch, had a negative effect (*P* < 0.001) on FI and BW gain. High lysine had a positive effect (*P* < 0.001) on BW gain and FI, and high lysine alleviated part of the detrimental effect of high AME from carbohydrates. This effect was visible from d 0 to d 11, and persisted till the end of the trial on d 35. In conclusion, feeding a diet with a high AME from carbohydrates has negative consequences for the development of the supply organs of broilers.

## INTRODUCTION

The metabolic state of broiler chickens fluctuates constantly due to varying nutrient requirements over time as a result of changes in maintenance and growth and dietary nutrient availability. At early ages, from hatch till d 7 or 8, supply organs (i.e., the gastrointestinal tract and associated organs, as well as respiratory and cardiovascular systems) have a high rate of allometric growth while maintenance requirements are relatively low (Iji et al. 2001a; Sklan, 2001). After the first week, the growth of several organs is isometric, whereas from d 17 or 18 onwards, the demand organs ([breast] muscle, skeleton, and adipose tissue) have a higher rate of allometric growth with increasing maintenance requirements (Iji et al., 2001b). To support the growth of demand organs, it is essential that the supply organs are appropriately developed to transport and provide the necessary nutrients. An inadequate development of the supply organs at a young age may therefore impact nutrient efficiency and hinder demand organs’ growth later.

To stimulate the optimal growth of the supply organs, there is a need for energy and protein (Nitsan et al., 1991). The lipid-rich residual yolk can be a temporary source of energy and early feed intake can provide the essential amino acids and remaining energy for growth. At hatch, broilers switch from relying on a lipid-rich yolk source to primarily utilizing carbohydrate-rich diets. This prompts a shift in the hormonal and nutrient-signaling pathways, from lipid oxidation to starch oxidation and glycolysis (Buyse et al., 2004). This abrupt change may take some time to adjust to for the post-hatch bird, potentially leading to incomplete breakdown and absorption processes from starch and high levels of incomplete glycolysis metabolites (e.g., upregulation of pyruvate kinase) (Honda et al., 2017). It can be hypothesized that this shift may lead to a temporary shortage of energy to support the supply organs’ growth.

Starch digestibility is relatively high compared to other nutrients in young broiler chickens (Sklan and Noy, 2000; Zelenka and Ceresnakova, 2005), which can be attributed to the rapid increase in amylase secretion following first feed intake after hatching (Noy and Sklan, 1995). The utilization of the resulting disaccharides, however, could be limited due to low mRNA activity of sucrase-isomaltase, l-aminopeptidase, ATPase, and the low numbers of sodium-glucose transporters in the small intestinal mucosa at hatch (Iji et al., 2001b; Uni et al., 2003b; Buyse et al., 2004). This means that while starch breakdown is shown to be high, disaccharides or glucose breakdown and absorption could still limit carbohydrate availability in young broilers. Most of the absorbed glucose is used for glycogen synthesis or non-essential amino acid production instead of oxidation (Buyse et al., 2004). Plasma glucose regulation, which is a function of age and diet in avian species, combined with strict homeostasis could also limit this process (Simon, 1989; Malheiros et al., 2003). An excess of glucose can decrease the release of glucagon from the α-cells and increase insulin from the β-cells of the pancreatic islets, resulting in increased glycogen synthesis in the hepatocytes through hexokinase IV and lower release of non-esterified fatty acids (Groop and Ferrannini, 1993; Printz et al., 1993a). This increased strain on the pancreas can lead to a higher risk of pancreatitis, and the higher glycogen synthesis in the liver leads to increased fat accumulation and fatty liver syndrome.

Aside from limitations of disaccharide digestibility and glycolysis, the post-enteral bioavailability of glucose depends on its metabolic fate during passage through the enterocytes. Under normal conditions, enterocytes mainly catabolize amino acids like glycine, glutamine, and glutamate to meet their energy demand (Watford, 2000). Glucose and some non-essential amino acids may compete for uptake in the small intestine, raising questions about which source is more efficiently absorbed and how this will impact enterocyte development.

The metabolic state of broiler chickens is, within certain boundaries, constantly fluctuating depending on age. In the early stages of life, supply organs undergo rapid growth, while facing a switch from lipid-rich to carbohydrate-rich energy sources. Although starch digestibility is high, subsequent steps and their impact on broiler chickens are not fully understood. The intricate mechanisms of supply organ development could be affected by an over- or undersupply of carbohydrates as an energy source. Therefore, the objective of this study was to determine if carbohydrates as an energy source at high or low amino acid density have a positive or negative impact on supply organ development.

## MATERIALS AND METHODS

All procedures in this study were approved by the Animal Welfare Committee of Schothorst feed research under number AVD246002016450, in accordance with Dutch laws and regulations on the execution of animal experiments.

### Experimental Design and Diets

This study was designed with 4 dietary treatments using a 2 × 2 factorial arrangement, with AME at 2 levels (E-: 2,750 kcal/kg and E+: 3,050 kcal/kg;) and standardized ileal digestible (**SID**) lysine at 2 levels (L-: 1.0% and L+: 1.2%) in the starter diets. The increase in AME was obtained by increasing maize starch, and digestible lysine by increasing; soybean meal, corn gluten meal and L-Lysine HCL. Available feed-grade synthetic amino acids were used to balance digestible amino acids in ratio to digestible lysine. The experimental starter diets (2.3 mm pellet; d 0–11) were followed by standard commercial grower (3.0 mm pellet; d 12–28; 3,000 kcal; 1.10% dig. Lys) and finisher diets (3.0 mm pellet; d 28–35; 3,100 kcal; 0.99% dig. Lys) for all treatments using similar raw materials, expect for corn starch, and according to breeder recommendations (Cobb-Vantress, 2018a). All diets were formulated based on digestibility, energy calculations, and nutrient data provided by Centraal Veevoeder Bureau (2016), with balancing ingredients such as; corn, wheat middlings, peas, potato protein, and corn gluten meal. Compositions of the starter diets are provided in Table 1; amino acids that were controlled in ratio to lysine are included. All diets were manufactured by ABZ De Samenwerking (Leusden, the Netherlands).

**Table 1:** Ingredient and nutrient composition of the experimental starter diets (d0–11).

Dietary energy	E-	E-	E+	E+
Dietary lysine	L-	L+	L-	L+
**Ingredient composition (g/kg)**				
Corn	334.2	364.3	330.3	235.4
Wheat	250.0	250.0	250.0	250.0
Soybean meal >48%CP	186.5	245.0	186.5	245.0
Wheat middlings	60.0	20.0	10.0	10.0
Peas	50.0	50.0	20.0	20.0
Potato protein	10.0	10.0	23.1	23.1
Corngluten meal 60%CP	5.0	5.0	15.0	50.0
Maize starch	5.0	5.0	100.0	100.0
Soyabean oil	13.0	13.0	13.0	13.0
Limestone	17.7	17.7	17.7	17.8
Monocalciumphosphate	7.7	7.7	8.6	8.7
Salt	1.4	1.4	1.4	1.3
Sodiumbicarbonate	3.3	3.3	3.3	3.3
L-lysine HCL	2.7	3.2	2.7	3.6
DL-methionine	2.4	3.1	2.4	2.6
L-threonine	1.0	1.2	0.9	1.1
L-valine	0.1	0.1	0.1	0.1
L-arginine	0.1	0.1	0.1	0.1
L-isoleucine	0.1	0.1	0.1	0.1
Premix starter	5.0	5.0	5.0	5.0
Phytase	3.3	3.3	3.3	3.3
Glucanase-Xylanase	2.5	2.5	2.5	2.5
Total	1,000.0	1,000.0	1,000.0	1,000.0
**Calculated chemical composition (g/kg)**				
Crude protein	185.0	215.0	185.0	215.0
Crude fat	34.1	34.9	34.6	35.3
Crude ash	56.5	56.9	52.7	52.9
Crude fiber	28.2	25.7	24.3	24.2
Dry matter	877.1	876.7	877.2	878.4
Starch (Ewers)	398.5	391.2	481.5	477.1
AME (kcal/kg)^1^	2750	2750	3050	3050
Digestible lysine^1^	10.0	12.0	10.0	12.0
Digestible methionine^1^	4.8	5.9	4.8	5.9
Digestible methionine & cysteine^1^	7.3	8.8	7.3	8.8
Digestible threonine^1^	6.5	7.8	6.5	7.8
Digestible tryptophan^1^	1.9	2.3	1.9	2.3
Digestible valine^1^	8.0	9.6	8.0	9.6
Digestible isoleucine^1^	6.6	8.0	6.6	8.0
Digestible arginine^1^	10.5	12.6	10.5	12.6
Digestible glycine & serine^1^	14.0	16.7	14.0	16.7
Digestible leucine^1^	12.7	15.3	12.7	15.3
Calcium	9.0	9.0	9.0	9.0
Phosphorus	5.7	5.3	5.1	5.1
**Analyzed chemical composition (g/kg; unless otherwise stated)**				
Crude protein	184	213	186	217
Crude fat	34	35	34	35
Dry matter	883	881	884	881
Starch	378	383	494	489
Calcium	8.8	9.1	8.7	8.8
Phosphorus	5.5	5.4	4.8	4.7
Sodium	1.4	1.5	1.5	1.4
Manganese (mg)	91	118	121	103

E- = Low dietary AME; E+ = High dietary AME; L- = Low digestible Lysine; L+ = High digestible Lysine.

1 Calculated according to CVB (2016).

All experimental diets were analyzed for dry matter (ISO 6496;1999), crude protein (ISO 16634-1;2008), crude fat by acid hydrolysis (ISO 6492;2002), ash (ISO 5984;2002), starch (ISO 6493;2000), and calcium, sodium, and manganese content by ICP-AES (ISO 27085;2009).

### Animals and Housing

In total, 3,200 eggs (weight range from 62 to 68 g) from a single-origin commercial Cobb 500 fast-feather flock were set for incubation at a commercial hatchery (Lagerwey, Lunteren, the Netherlands). Eggs were incubated according to the breed standards (Cobb-Vantress 2018b). At 12 h before the estimated hatch time the first 600 to 620 hatched chickens were removed from the hatching baskets. Around 75% of the total hatch, 1,800 freshly hatched broiler chickens were selected and brought to a separate room for sexing and selection for the experiment. Female broiler chickens were excluded from the experiment. The 800 selected male broiler chickens were transported to the facility of Schothorst feed research (Lelystad, the Netherlands), and distributed over 40 pens (1.00 × 2.00 m) with 20 broilers per pen. Floors were covered with fresh wood shavings and each pen had a round feeder and 4 nipple drinkers. Treatments were randomly assigned to the pens within 10 blocks, resulting in 10 replications per treatment. The average chick weight was determined on arrival at the facility. Afterward, 20 broiler chickens were randomly selected and placed in a pen. Initial pen weight was checked to be within +1 or -1 standard deviation of the average estimated pen weight. Feed and water were available ad libitum from the moment of placement until the end of the experiment. Temperature and lighting were set to breeder recommendations during the entire grow-out period (Cobb-Vantress, 2018a). A caretaker checked the animals twice per day. Mortality was recorded daily.

### Data Collection

The BW and feed intake (**FI**) of the chickens were measured per pen on d 0, 4, 11, 28, and 35. The gain ratio (**F:G**) was calculated based on FI and BW gain. Nutrient intake was determined by multiplying FI with the calculated nutrient content of the diet, and BW gain per nutrient was calculated using the nutrient intake. Before placement, 20 broiler chickens were randomly selected and during the trial a random broiler chicken from each pen was selected, weighed, and euthanized by CO_2_ on d 0, d 4, and 11. Immediately after euthanasia, a blood sample was collected, and the total carcass was weighed after bleeding. The carcass was dissected to determine the weight of the heart, proventriculus, gizzard, liver, pancreas, duodenum, jejunum, ileum, ceca, colon, yolk sac, and the remainder of the carcass (including head, feathers, and all other parts not dissected). Before the proventriculus and gizzard were weighed, they were cut open and rinsed to remove feed residuals. The small intestines, ceca, and colon were emptied by gentle squeezing before they were weighed. The length of the duodenum (duodenal loop excluding pancreas), jejunum (from the end of the duodenum to Meckel's diverticulum), ileum (from Meckel's diverticulum to ileal-cecal junction), ceca and colon were measured.

### Histology

Samples from the middle of the duodenum, jejunum, and ileum were taken during dissection on d 4 and 11. After the middle point of each section was determined, approximately 1 mL formaldehyde 10% solution was injected for flushing at 5 cm from the middle point. The section was excised and stored in a 10% formaldehyde solution at room temperature. Samples were embedded in paraffin and cut into 5 to 6 µm sections using a microtome (Microm HM355S; Thermo Fisher Scientific, Waltham, MA), and mounted on glass slides.

Samples were hematoxylin and eosin (**HE**) stained for determination of villus length, crypt depth, and goblet cell count, using cellSens Dimensions (Olympus; Tokyo, Japan). Cells expressing mucin glycoprotein were identified using a combined alcian blue (AB) and periodic acid Schiff staining, as described by (Layton et al., 2019).

### Allometric Growth Calculation

For allometric growth, Huxley's allometric growth model (Huxley and Teissier, 1936) was used. Growth curves were estimated for each treatment to predict heart, liver, proventriculus, gizzard, pancreas, duodenum, jejunum, ileum, and ceca weight using the equation:

$$y=ax^{b},$$

where *y* is the weight of organ (in g), *x* is yolk-free BW (in g), and *a* and *b* are coefficients estimated using the NLIN procedure in SAS (Version 9.3, 2011, SAS Institute Inc., Cary, NC, United States). Yolk-free BW was used given the impact on d 0 on organ weights in comparison to total BW, but using yolk-free BW had limited influence on d 4 and none on d 11. The values for *a* are considered the intercept or scale variable. Values for *b* determine the growth rate of organs compared to the body as a whole; <1 is a slower growth rate, =1 is an equal growth rate (isometry), and >1 is a faster growth rate. Curves were estimated using data from d 0 and 4, d 0 and 11, d 4 and 11, and the overall period of d 0, 4, and 11.

### Statistical Analysis

Data were subjected to 2-way ANOVA, using the PROC GLM procedure in SAS (Version 9.3, 2011, SAS Institute Inc., Cary, NC). Mortality data were analyzed as binomially distributed data, using the same statistical model. Data are expressed as least square **(***LS***)** means ± SEM. Effects were considered significant when *P* ≤ 0.05 and a trend when 0.05 < *P* ≤ 0.10.

The allometric growth curves were compared between treatments and age ranges using nonlinear analysis of covariance. For this, the null hypothesis of equality was evaluated using a paired F-test procedure described by (Motulsky and Ransnas, 1987). Treatments (***trt***) were compared in pairs by estimating the curves for each, using the NLIN procedure of SAS. From this analysis the overall sum of squares for the separate fits (*SS_sep_*) were calculated (*SS_sep_ = SS_trt1_ + SS_trt2_*), as well as the total number of degrees of freedom (*df*) (*df_sep_ = df_trt1_ + df_trt2_*). After this, data for both treatments were pooled and a single allometric curve was estimated, and the pooled total sum of squares (*SS_pool_*) and degrees of freedom (*df_pool_*) were determined. Next, the F ratio was calculated:

$$F=\frac{\left(SS_{pool}-SS_{sep}\right)/\left(df_{pool}-df_{sep}\right)}{SS_{sep}/df_{sep}}.$$

A large F value (low *P*-value) indicated that the variation was better explained in separate fits compared to the pooled fit. When *P* ≤ 0.05, the null hypothesis was rejected and the allometric curves were considered different.

## RESULTS

### Growth Performance and Nutrient Intake

The effects of the starter diet on performance from d 0 till 35 are shown for each feeding phase in Table 2. An interaction between AME and lysine was found for BW from d 4 to 35 and BW gain from d 0 to 28. Notably, the combination of high AME (E+) and high lysine (L+) resulted in higher BW gain from d 0 to 4 compared to low AME (E-) with low lysine (L-) and E+L-. Moreover, from d 4 to 11, BW gain was highest in E-L+, whereas E+L- yielded the lowest gain. By d 11, this translated into the highest BW in E-L+, contrasting with the lowest BW in E+L-. This interaction effect on BW gain persisted until d 28, and for BW until d 35, culminating in the lowest BW on d 35 for E+L-, a higher BW for E-L- and E+L+, and the highest BW for E-L+.

**Table 2:** Effects of dietary AME from carbohydrates and digestible lysine in the starter phase on body weight, body weight gain, feed intake and feed to gain ratio.^1^

	E		L		E x L					*P*-values		
	E-	E+	L-	L+	E-L-	E-L+	E+L-	E+L+	SEM	E	L	E x L
n	20	20	20	20	10	10	10	10				
**BW (g)**												
0 d	45.7	45.7	45.7	45.7	45.7	46	45.9	45.6	0.1	0.991	0.754	0.598
4 d	107.9	107.6	105.2^y^	110.3^x^	105.3^c^	109.6^b^	105.0^c^	110.4^a^	0.3	0.526	<0.001	0.003
11 d	348.3^A^	332.1^B^	324.4^y^	356.0^x^	329.5^c^	365.1^a^	321.1^d^	348.4^b^	2.9	<0.001	<0.001	<0.001
28 d	1840.6^A^	1762.3^B^	1752.5^y^	1850.3^x^	1801.5^b^	1876.1^a^	1725.2^c^	1818.4^b^	11.6	<0.001	<0.001	<0.001
35 d	2651.3^A^	2548.3^B^	2546.9^y^	2652.6^x^	2616.1^b^	2701.2^a^	2539.0^c^	2612.4^b^	17.3	0.004	<0.001	0.013
**BW gain (g/bird)**												
0–4 d	61.6	61.4	58.9^y^	64.2^x^	59.6^b^	63.6^a^	58.1^c^	64.7^a^	0.2	0.471	<0.001	<0.001
4–11 d	241^A^	227^B^	219^y^	247^x^	224^c^	255^a^	216^d^	238^b^	2.4	<0.001	<0.001	<0.001
0–11 d	303^A^	286^B^	279^y^	310^x^	284^c^	319^a^	274^d^	303^b^	2.9	<0.001	<0.001	<0.001
11–28 d	1514^A^	1438^B^	1438^y^	1510^x^	1472^b^	1551^a^	1404^c^	1470^b^	9.0	<0.001	<0.001	<0.001
28–35 d	820	804	815	810	815	825	814	794	4.1	0.054	0.290	0.143
0–35 d	2612^A^	2531^B^	2531^y^	2611^x^	2570	2655	2492	2567	16.7	0.007	<0.001	0.678
**FI (g/bird)**												
0-4 d	57.6	58.9	56.5^y^	60.2^x^	56.7^c^	58.2^b^	56.2^c^	61.9^a^	0.2	0.574	0.004	0.037
4-11 d	289^A^	272^B^	276^y^	284^x^	286^ab^	291^a^	267^c^	277^b^	1.9	<0.001	<0.001	<0.001
0-11 d	346^A^	330^B^	333^y^	343^x^	342	349	322	338	2.6	<0.001	0.016	0.052
11-28 d	2084	2012	1997^y^	2100^x^	2053^b^	2119^a^	1940^c^	2082^b^	11.2	0.053	<0.001	<0.001
28-35 d	1288	1275	1278	1286	1283	1290	1273	1279	9.6	0.674	0.687	0.452
0-35 d	3718^A^	3618^B^	3607^y^	3729^x^	3678	3758	3536	3700	18.5	0.001	0.001	0.062
**F:G (g:g)**												
0–4 d	0.930	0.965	0.956	0.939	0.945	0.918	0.967	0.961	0.010	0.681	0.390	0.248
4–11 d	1.206	1.199	1.256^x^	1.151^y^	1.274^a^	1.138^d^	1.235^b^	1.165^c^	0.009	0.186	<0.001	<0.001
0–11 d	1.148	1.145	1.189^x^	1.106^y^	1.203^a^	1.093^b^	1.175^ab^	1.117^b^	0.009	0.399	<0.001	<0.001
11–28 d	1.398	1.400	1.387	1.409	1.394	1.403	1.380	1.417	0.005	0.913	0.115	0.273
28–35 d	1.568	1.589	1.569	1.588	1.574	1.562	1.564	1.611	0.028	0.416	0.233	0.224
0–35 d	1.425	1.429	1.425	1.428	1.431^ab^	1.414^b^	1.418^b^	1.442^a^	0.006	0.441	0.141	0.046

E = Dietary AME; E- = Low dietary AME; E+ = High dietary AME; L = Digestible Lysine; L- = Low digestible Lysine; L+ = High digestible Lysine.

1 Data are means.

A,B Values within a row with different superscript differ significantly at *P*≤ 0.05 on E

x,y Values within a row with different superscript differ significantly at *P*≤ 0.05 on L

a-d Values within a row with different superscript differ significantly at *P*≤ 0.05 on the interaction between E and L.

Furthermore, an interaction between AME and lysine was found for FI from d 0 to 28 and F:G ratio from d 4 to 11 and throughout the overall period. Notably, the combination of E+L+ led to the highest FI from d 0 to 4, while E-L- and E+L- resulted in the lowest FI. From d 4 to 11 and d 11 to 28 FI was highest in E-L+, whereas E+L- resulted in the lowest FI. This trend of interaction persisted throughout the grower period till d 28. In terms of the F:G ratio, no main or interaction effects were observed from d 0 to 4. However, E-L+ resulted in the lowest F:G ratio from d 4 to 11, while E-L- had the highest F:G ratio. Over the entire period, the F:G ratio was highest in E+L+, whereas E-L+ and E+L- resulted in the lowest F:G ratio.

The intake of nutrients and gain per nutrient are shown in Table 3 for d 0 to 4 and d 4 to 11. An interaction between AME and lysine was found for AME and fat intake. AME intake from d 0 to 4 was highest in E+L+ compared to the other treatments, with E+L+ maintaining the highest intake from d 4 to 11, while E+L- had the lowest. Similarly, fat intake from d 0 to 4 was highest in E+L+ and lowest in E+L-, whereas from d 4 to 11, fat intake was highest in E-L+ and still lowest in E+L-. Diets high in lysine resulted in an increased lysine intake from d 0 to 4 and d 4 to 11, while high AME diets resulted in a higher starch intake in the same periods.

**Table 3:** Effects of dietary AME from carbohydrates and digestible Lysine in the starter phase on the nutrient intake and nutrient gain ratio.^1^

	E		L		E x L					*P*-values		
	E-	E+	L-	L+	E-L-	E-L+	E+L-	E+L+	SEM	E	L	E x L
n	20	20	20	20	10	10	10	10				
**Nutrient intake**												
**AME (Kcal/bird)**												
0–4 d	165.3^B^	178.4^A^	164.6^y^	178.8^x^	162.2^b^	168.5^b^	167.1^b^	189.4^a^	2.7	0.046	0.031	0.001
4–11 d	827.2	820.3	811.3^y^	836.1^x^	823.0^b^	831.1^ab^	799.5^c^	841.2^a^	3.1	0.101	<0.001	<0.001
**Lys (g)**												
0–4 d	0.64	0.65	0.56^y^	0.73^x^	0.57	0.71	0.55	0.75	0.02	0.584	<0.001	0.699
4–11 d	3.17	2.97	2.76^y^	3.39^x^	2.87	3.48	2.64	3.30	0.12	0.487	<0.001	0.564
**Starch (g/bird)**												
0–4 d	22.7^B^	28.4^A^	24.8	26.2	22.6	22.8	27.1	29.5	1.8	<0.001	0.643	0.263
4–11 d	113.8^B^	130.1^A^	121.0	122.8	113.8	113.8	128.3	131.9	1.6	<0.001	0.712	0.297
**Fat (g/bird)**												
0–4 d	1.97^B^	2.06^A^	1.91^y^	2.12^x^	1.91^c^	2.04^b^	1.92^c^	2.19^a^	0.01	<0.001	<0.001	<0.001
4–11 d	9.98^A^	9.46^B^	9.45^y^	10.00^x^	9.74^b^	10.21^a^	9.16^c^	9.78^b^	0.07	<0.001	<0.001	<0.001
**Nutrient gain ratio**												
**AME:G (Kcal:g)**												
0–4 d	2.70^B^	2.89^A^	2.80	2.78	2.73	2.68	2.87	2.90	0.03	<0.001	0.356	0.121
4–11 d	3.39^B^	3.66^A^	3.71^x^	3.32^y^	3.62^c^	3.16^d^	3.81^a^	3.49^b^	0.03	<0.001	<0.001	<0.001
**Lys:G (mg:g)**												
0–4 d	1.024	1.032	0.940^y^	1.117^x^	0.938	1.112	0.944	1.119	0.006	0.205	<0.001	0.101
4–11 d	1.295^B^	1.331^A^	1.249^y^	1.376^x^	1.236^d^	1.355^b^	1.267^c^	1.394^a^	0.005	<0.001	<0.001	<0.001
**Starch:G (g:g)**												
0–4 d	0.36^B^	0.40^A^	0.39	0.36	0.36^c^	0.35^c^	0.42^a^	0.39^b^	0.01	<0.001	0.110	<0.001
4–11 d	0.45^B^	0.52^A^	0.53	0.47	0.49^b^	0.43^c^	0.56^a^	0.48^b^	0.01	<0.001	0.102	<0.001
**Fat:G (g:g)**												
0–4 d	0.044	0.040	0.044	0.040	0.049^a^	0.037^c^	0.039^bc^	0.042^b^	0.001	0.214	0.307	<0.001
4–11 d	0.056	0.051	0.058	0.050	0.066^a^	0.045^c^	0.051^b^	0.052^b^	0.001	0.493	0.291	<0.001

E = Dietary AME; E- = Low dietary AME; E+ = High dietary AME; L = Digestible Lysine; L- = Low digestible Lysine; L+ = High digestible Lysine.

1 Data are means.

A,B Values within a row with different superscript differ significantly at *P*≤ 0.05 on E

x,y values within a row with different superscript differ significantly at *P*≤ 0.05 on L

a-d values within a row with different superscript differ significantly at *P*≤ 0.05 on the interaction between E and L.

Moreover, an interaction between AME and lysine was found for the nutrient-to-gain ratio of AME, lysine, starch, and fat. Specifically, the AME:gain ratio was highest in E+L- from d 4 to 11 and lowest in E-L+, whereas the lys:gain ratio was highest in E+L+ and lowest in E-L- during the same period. The starch:gain ratio was highest in E+L- from d 0 to 4 and d 4 to 11, while lowest in E-L+ for both periods. Lastly, the fat:gain ratio was highest in E-L- from d 0 to 4 and d 4 to 11, whereas it was lowest in E-L+.

### Supply Organ Weight and Histo-Morphological Parameters

The effects of the starter diet on relative organ weights are shown in Table 4, Table 5 for d 4 and d 11, respectively, while the length and relative weight of the small intestine for both days are presented in Table 6. Additionally, the BW of the dissected birds is reported herein, demonstrating similarity to the average BW of the treatment group on both days. An interaction between AME and lysine was found for relative liver weight, relative proventriculus weight, and relative ileum weight. Specifically, the relative liver weight was highest in the E+L- on d 4 and 11, while it was lowest in E-L- on d 4 and E-L+ on d 11. On d 4, relative proventriculus weight was highest in E-L+ and lowest in E+L-, decreasing with increased AME. However, on d 11 relative proventriculus weight decreased with both increased AME and Lys, lowest in E+L+ and highest in E-L-. The relative weight of the ileum responded similarly for both d 4 and 11, decreasing with increased AME and Lys, lowest in E+L+ and highest in E-L-. Notably, the relative weight difference between E-L- and E-L+ was smaller compared to E+L- and E+L-. A trend was observed on d 11 of a lower relative pancreas weight with higher lysine. Relative weights of all other organs remained similar across treatments.

**Table 4:** Effects of dietary AME from carbohydrates and digestible lysine in the starter phase on supply organ weight as percentage of bodyweight at d 4.^1^

	E		L		E x L					*P*-values		
	E-	E+	L-	L+	E-L-	E-L+	E+L-	E+L+	SEM	E	L	E x L
n	20	20	20	20	10	10	10	10				
**Live BW (g)**	116.5	116.6	113.1^y^	120.2^x^	113.9^ab^	119.2^ab^	112.2^b^	120.9^a^	2.1	0.964	<0.001	0.009
**Supply organ weight d 4, % of BW**												
Heart	0.96	0.93	0.93	0.95	0.94	0.97	0.94	0.92	0.10	0.556	0.940	0.862
Liver	4.92^B^	5.46^A^	5.10	5.29	4.60^b^	5.24^ab^	5.57^a^	5.34^ab^	0.10	0.007	0.348	0.008
Proventriculus	1.14^A^	0.98^B^	1.05	1.06	1.13^a^	1.14^a^	0.96^c^	0.99^b^	0.01	0.001	0.663	0.008
Gizzard	4.35	4.28	4.39	4.25	4.56	4.14	4.21	4.36	0.23	0.516	0.617	0.345
Pancreas	0.42	0.44	0.43	0.43	0.40	0.43	0.45	0.42	0.02	0.421	0.970	0.423
Duodenum	1.79	1.71	1.78	1.70	1.80	1.77	1.77	1.65	0.06	0.217	0.450	0.486
Jejunum	3.47	3.26	3.36	3.39	3.40	3.56	3.31	3.21	0.19	0.135	0.773	0.491
Ileum	2.59	2.24	2.53	2.29	2.63^a^	2.54^a^	2.42^ab^	2.03^b^	0.07	0.120	0.055	0.015
Ceca	0.75	0.72	0.74	0.73	0.72	0.77	0.75	0.70	0.03	0.421	0.691	0.867

E = Dietary AME; E- = Low dietary AME; E+ = High dietary AME; L = Digestible Lysine; L- = Low digestible Lysine; L+ = High digestible Lysine.

1 Data are means.

A,B Values within a row with different superscript differ significantly at *P*≤ 0.05 on E

x,y values within a row with different superscript differ significantly at *P* ≤ 0.05 on L

a-d values within a row with different superscript differ significantly at *P* ≤ 0.05 on the interaction between E and L.

**Table 5:** Effects of dietary AME from carbohydrates and digestible lysine in the starter phase on supply organ weight as percentage of body weight at d 11.^1^

	E		L		E x L					*P-*values		
	E-	E+	L-	L+	E-L-	E-L+	E+L-	E+L+	SEM	E	L	E x L
n	20	20	20	20	10	10	10	10				
**BW (g)**	363.1	344.8	331.3^y^	376.7^x^	341.7	384.7	320.9	368.4	11.2	0.308	<0.001	0.170
**Supply organ weight d 11, % of BW**												
Heart	0.75	0.78	0.77	0.76	0.77	0.73	0.77	0.79	0.10	0.296	0.736	0.597
Liver	4.09^B^	4.69^A^	4.45	4.32	4.11	4.06	4.77	4.58	0.16	<0.001	0.178	0.181
Proventriculus	0.76^A^	0.70^B^	0.77^x^	0.69^y^	0.79^a^	0.72^b^	0.74^ab^	0.68^b^	0.02	0.050	0.003	0.003
Gizzard	2.97	2.97	3.05	2.89	3.11	2.82	2.98	2.96	0.12	0.890	0.185	0.476
Pancreas	0.44	0.43	0.45	0.42	0.46	0.41	0.44	0.41	0.02	0.284	0.057	0.172
Duodenum	1.42	1.50	1.49	1.42	1.44	1.39	1.54	1.45	0.09	0.432	0.400	0.549
Jejunum	2.61	2.55	2.65	2.50	2.65	2.57	2.65	2.45	0.10	0.427	0.281	0.529
Ileum	1.97	1.82	1.97^x^	1.80^y^	2.03^a^	1.88^ab^	1.90^ab^	1.73^b^	0.04	0.064	0.040	0.049
Ceca	0.60	0.62	0.62	0.60	0.61	0.59	0.63	0.60	0.08	0.813	0.504	0.881

E = Dietary AME; E- = Low dietary AME; E+ = High dietary AME; L = Digestible Lysine; L- = Low digestible Lysine; L+ = High digestible Lysine.

1 Data are means

A,B Values within a row with different superscript differ significantly at *P* ≤ 0.05 on E

x,y values within a row with different superscript differ significantly at *P*≤ 0.05 on L

a-d values within a row with different superscript differ significantly at *P* ≤ 0.05 on the interaction between E and L.

**Table 6:** Effects of dietary AME from carbohydrates and digestible lysine in the starter phase on length and weight to length ratio of the small intestine on d 4 and 11.^1^

	E		L		E x L					*P*-values		
	E-	E+	L-	L+	E-L-	E-L+	E+L-	E+L+	SEM	E	L	E x L
n	20	20	20	20	10	10	10	10				
**D 4**												
**Duodenum**												
Length (cm)	14.4	14.2	14.3	14.3	14.5	14.2	14.1	14.3	0.2	0.846	0.794	0.882
g/cm	0.147	0.136	0.141	0.142	0.145	0.149	0.139	0.135	0.003	0.109	0.635	0.424
**Jejunum**												
Length (cm)	32.6	32.0	32.2	32.4	32.9	32.3	31.5	32.5	0.5	0.317	0.164	0.132
g/cm	0.124	0.118	0.119	0.124	0.120	0.129	0.117	0.118	0.003	0.451	0.246	0.281
**Ileum**												
Length (cm)	33.0^A^	30.9^B^	32.2	31.7	32.6^a^	33.4^a^	31.6^ab^	30.1^b^	0.3	0.042	0.059	0.026
g/cm	0.091	0.087	0.090	0.086	0.093	0.088	0.087	0.084	0.004	0.173	0.104	0.087
**D 11**												
**Duodenum**												
Length (cm)	10.8	10.7	10.7	10.8	11.1	10.4	10.3	11.1	0.2	0.897	0.722	0.673
g/cm	0.485	0.478	0.463	0.500	0.451	0.519	0.475	0.481	0.011	0.282	0.163	0.058
**Jejunum**												
Length (cm)	47.6	47.9	46.5	49.1	45.3	50.0	47.7	48.1	0.6	0.411	0.055	0.092
g/cm	0.203	0.185	0.193	0.195	0.207	0.198	0.178	0.192	0.009	0.243	0.307	0.121
**Ileum**												
Length (cm)	48.3	48.2	47.3	49.1	48.7^ab^	47.8^ab^	46.0^b^	50.4^a^	0.3	0.085	0.052	0.031
g/cm	0.151^A^	0.132^B^	0.140	0.141	0.149	0.153	0.133	0.129	0.011	0.006	0.714	0.267

E = Dietary AME; E- = Low dietary AME; E+ = High dietary AME; L = Digestible Lysine; L- = Low digestible Lysine; L+ = High digestible Lysine.

1 Data are means.

A,B Values within a row with different superscript differ significantly at *P*≤ 0.05 on E ^x,y^values within a row with different superscript differ significantly at *P*≤ 0.05 on L

a-d values within a row with different superscript differ significantly at *P*≤ 0.05 on the interaction between E and L..

Furthermore, an interaction between AME and lysine was found for ileum length on d 4 and 11. Specifically, on d 4, E-L- resulted in a longer ileum and a trend of higher weight per cm, while E+L+ resulted in a shorter ileum. Conversely, on d 11, E+L+ resulted in a longer ileum, while E+L- resulted in a shorter ileum. Moreover, the weight per cm of ileum was lower with high AME on d 11. Additionally, the length of the jejunum showed a trend towards being higher with E-L+ compared to E-L- on d 11. The duodenum was not affected by AME or lysine.

The results of villus length and crypt depth are shown in Table 7, Table 8 for d 4 and 11, respectively. Duodenal villus length was higher in the low AME groups on both d 4 and 11. An interaction between AME and lysine was found for jejunal villus length and crypt depth on d 4. Villus length was higher with deeper crypts in E+L+, while villus length was shorter with lower crypt depth in E+L-; however, the villus:crypt ratio remained similar. On d 11, a trend towards higher jejunal villus length with increased AME was observed. There were no differences found in the total number of goblet cells, number of goblet cells with acidic mucus, goblet cell size, or area of the goblet cells per villus (see Table 9, Table 10)

**Table 7:** Effects of dietary AME from carbohydrates and digestible lysine in the starter phase on villus length, crypt depth, and villus to crypt ratio at d 4.^1^

	E		L		E x L					*P*-values		
	E-	E+	L-	L+	E-L-	E-L+	E+L-	E+L+	SEM	E	L	E x L
n	20	20	20	20	10	10	10	10				
**Duodenum**												
Villus length (µm)	1053^A^	950^B^	999	1005	1048	1058	949	951	27	0.003	0.851	0.909
Crypt depth (µm)	166	158	162	162	169	162	154	161	8	0.188	0.990	0.279
V:C ratio	6.6	6.3	6.4	6.4	6.4	6.7	6.4	6.1	0.4	0.279	0.862	0.302
**Jejunum**												
Villus length (µm)	518	505	490	533	531^ab^	504^b^	448^c^	561^a^	15	0.645	0.117	0.013
Crypt depth (µm)	138	135	135	139	144^ab^	132^bc^	125^c^	145^a^	3	0.521	0.455	0.004
V:C ratio	3.9	3.8	3.8	3.9	3.8	3.9	3.7	3.9	0.1	0.772	0.423	0.793
**Ileum**												
Villus length (µm)	401	376	394	383	406	396	382	369	14	0.131	0.793	0.923
Crypt depth (µm)	127	120	122	124	123	130	121	118	5	0.237	0.793	0.546
V:C ratio	3.3	3.3	3.4	3.2	3.4	3.1	3.3	3.2	0.2	0.981	0.247	0.678

E = Dietary AME; E- = Low dietary AME; E+ = High dietary AME; L = Digestible Lysine; L- = Low digestible Lysine; L+ = High digestible Lysine.

1 Data are means.

A,B Values within a row with different superscript differ significantly at *P*≤ 0.05 on E ^x,y^values within a row with different superscript differ significantly at *P*≤ 0.05 on L

a-d values within a row with different superscript differ significantly at *P*≤ 0.05 on the interaction between E and L.

**Table 8:** Effects of dietary AME from carbohydrates and digestible lysine in the starter phase on villus length, crypt depth, and villus to crypt ratio at d 11^1^.

	E		L		E x L					*P*-values		
	E-	E+	L-	L+	E-L-	E-L+	E+L-	E+L+	SEM	E	L	E x L
n	20	20	20	20	10	10	10	10				
**Duodenum**												
Villus length (µm)	1523^A^	1438^B^	1484	1478	1534	1512	1433	1443	22	0.003	0.929	0.803
Crypt depth (µm)	276	261	266	271	270	282	262	260	9	0.173	0.686	0.520
V:C ratio	5.7	5.7	5.7	5.6	5.8	5.5	5.6	5.7	0.3	0.984	0.737	0.485
**Jejunum**												
Villus length (µm)	876	923	895	904	862	889	927	919	19	0.081	0.818	0.695
Crypt depth (µm)	228	244	241	230	240	215	242	245	12	0.198	0.561	0.159
V:C ratio	4.0	3.9	3.9	4.0	3.8	4.2	4.0	3.8	0.2	0.585	0.462	0.170
**Ileum**												
Villus length (µm)	566	538	552	552	566	565	537	539	14	0.435	0.859	0.890
Crypt depth (µm)	189	188	182	195	178	200	186	189	10	0.859	0.167	0.380
V:C ratio	3.1	3.0	3.1	2.9	3.2	2.9	3.0	2.9	0.2	0.414	0.108	0.245

E = Dietary AME; E- = Low dietary AME; E+ = High dietary AME; L = Digestible Lysine; L- = Low digestible Lysine; L+ = High digestible Lysine.

1 Data are means.

A,B Values within a row with different superscript differ significantly at *P*≤ 0.05 on E; ^x,y^values within a row with different superscript differ significantly at *P*≤ 0.05 on L; ^a-d^ values within a row with different superscript differ significantly at *P*≤ 0.05 on the interaction between E and L.

**Table 9:** Effects of dietary AME from carbohydrates and digestible lysine in the starter phase on goblet cell development on d 4^1^.

	E		L		E x L					*P*-values		
	E-	E+	L-	L+	E-L-	E-L+	E+L-	E+L+	SEM	E	L	E x L
n	16	16	16	16	8	8	8	8				
**Duodenum**												
Total goblet cells per villus	80	78	85	73	82	77	88	68	4.4	0.876	0.153	0.387
Acidic goblet cells per villus	353	63	357	59	645	61	69	56	4.6	0.749	0.127	0.416
Goblet cells surface per 1mm of villus	1921	1852	2103	1672	2028	1817	2179	1525	129	0.784	0.100	0.394
Goblet cell size (µm)	24	23	24	23	24	23	24	22	0.5	0.759	0.271	0.446
Goblet cell surface as % of villus	1.34	1.37	1.49	1.22	1.45	1.24	1.54	1.20	0.09	0.874	0.128	0.728
**Jejunum**												
Total goblet cells per villus	55	54	50	59	54	56	46	62	2.9	0.874	0.131	0.211
Acidic goblet cells per villus	44	43	40	47	42	45	37	49	3.1	0.793	0.355	0.427
Goblet cells surface per 1mm of villus	1347	1229	1193	1380	1355	1338	1030	1424	88	0.501	0.294	0.251
Goblet cell size (µm)	24	23	23	24	24	24	22	23	0.7	0.465	0.974	0.594
Goblet cell surface as % of villus	2.08	1.85	1.91	2.00	2.13	2.02	1.68	2.00	0.14	0.407	0.726	0.454
**Ileum**												
Total goblet cells per villus	60	61	60	61	61	58	58	63	3.3	0.918	0.928	0.536
Acidic goblet cells per villus	47	47	48	46	50	43	45	49	3.3	0.893	0.919	0.663
Goblet cells surface per 1mm of villus	1251	1245	1166	1330	1167	1334	1164	1325	66	0.965	0.229	0.980
Goblet cell size (µm)	23	22	22	23	21	24	22	22	1.1	0.834	0.545	0.568
Goblet cell surface as % of villus	2.87	3.03	2.80	3.12	2.71	3.02	2.86	3.21	0.15	0.585	0.284	0.940

E = Dietary AME; E- = Low dietary AME; E+ = High dietary AME; L = Digestible Lysine; L- = Low digestible Lysine; L+ = High digestible Lysine.

1 Data are means. ^A,B^Values within a row with different superscript differ significantly at *P*≤ 0.05 on E; ^x,y^values within a row with different superscript differ significantly at *P*≤ 0.05 on L; ^a-d^values within a row with different superscript differ significantly at *P*≤ 0.05 on the interaction between E and L.

**Table 10:** Effects of dietary AME from carbohydrates and digestible lysine in the starter phase on goblet cell development on d 11.^1^

	E		L		E x L					*P*-values		
	E-	E+	L-	L+	E-L-	E-L+	E+L-	E+L+	SEM	E	L	E x L
n	16	16	16	16	8	8	8	8				
**Duodenum**												
Total goblet cells per villus	174	173	170	178	160	188	179	167	8.3	0.946	0.643	0.244
Acidic goblet cells per villus	125	128	124	129	116	134	131	124	7.6	0.917	0.511	0.393
Goblet cells surface per 1mm of villus	4159	4311	4064	4405	3665	4652	4462	4158	253	0.772	0.516	0.223
Goblet cell size (µm)	23	25	24	25	22	24	25	25	0.7	0.256	0.388	0.358
Goblet cell surface as % of villus	1.60	1.70	1.60	1.69	1.42	1.77	1.78	1.59	0.09	0.617	0.661	0.154
**Jejunum**												
Total goblet cells per villus	139	142	138	143	130	148	146	138	9.4	0.881	0.837	0.516
Acidic goblet cells per villus	105	104	102	107	96	114	108	99	8.3	0.876	0.799	0.548
Goblet cells surface per 1mm of villus	2988	3254	3090	3150	2655	3321	3529	2978	240	0.592	0.907	0.224
Goblet cell size (µm)	22	23	22	22	21	22	23	22	0.6	0.340	0.960	0.369
Goblet cell surface as % of villus	2.16	2.07	2.14	2.10	1.95	2.39	2.34	1.80	0.12	0.701	0.776	0.051
**Ileum**												
Total goblet cells per villus	115	124	128	111	132	98	124	124	7.1	0.552	0.233	0.223
Acidic goblet cells per villus	86	96	95	86	96	75	94	97	6.8	0.471	0.275	0.273
Goblet cells surface per 1 mm of villus	1708	1603	1802	1509	1852	1564	1752	1454	114	0.654	0.216	0.983
Goblet cell size (µm)	16	14	15	15	15	17	15	13	0.9	0.354	0.922	0.221
Goblet cell surface as % of villus	2.29	2.32	2.48	2.15	2.40	2.17	2.58	2.08	0.13	0.863	0.181	0.624

E = Dietary AME; E- = Low dietary AME; E+ = High dietary AME; L = Digestible Lysine; L- = Low digestible Lysine; L+ = High digestible Lysine.

1 Data are means. ^A,B^Values within a row with different superscript differ significantly at *P*≤ 0.05 on E; ^x,y^values within a row with different superscript differ significantly at *P*≤ 0.05 on L; ^a-d^values within a row with different superscript differ significantly at *P*≤ 0.05 on the interaction between E and L.

### Allometric Growth

**Figure 1:**
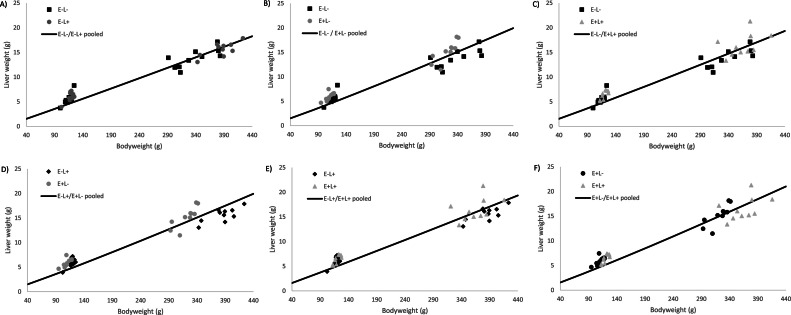
Treatment comparison of allometric growth curves of the liver using data from d 0, 4 and 11. The line shows the pooled allometric growth of 2 treatments, and the symbols represent measured liver weights. Graphs B, D, and F show that the separate fits explain the variation better, compared to the pooled fit. Whereas, graphs A, C, and E show the separate fits and pooled fit both explain the variation.

Allometric growth coefficients for the supply organs are shown in the Table 11, and results for the equality of the null hypothesis can be found in Table 12. The heart exhibited a higher allometric growth rate in the E+L+ group from d 4 onwards compared to the other groups. The liver allometric growth rate was higher in the high AME groups in the period from d 0 to 4 and d 0 to 11, with a higher allometric growth rate from d 4 to 11 in the high lysine groups. When considering all data points, the allometric growth rate was higher in the E+L- compared to the other groups (see Figure 1).

**Table 11:** Allometric growth coeffcients^1^ for effects of AME from carbohydrates and digestible lysine in the starter phase on supply organs at d 0 and 4, d 0 and 11, d 4 and 11, and d 0 and 4 and 11.

		D 0 and 4		D 0 and 11		D 4 and 11		D 0 and 4 and 11	
Organ	Diet treatment	a	b	a	b	a	b	a	b
Heart	E-L-	0.005	1.129	0.011	0.942	0.026	0.790	0.013	0.903
	E-L+	0.005	1.121	0.010	0.947	0.024	0.800	0.014	0.896
	E+L-	0.005	1.121	0.010	0.944	0.025	0.792	0.013	0.905
	E+L+	0.006	1.112	0.009	0.980	0.020	0.845	0.012	0.931
Liver	E-L-	0.003	1.580	0.015	1.167	0.096	0.854	0.031	1.048
	E-L+	0.003	1.594	0.015	1.164	0.123	0.814	0.037	1.019
	E+L-	0.002	1.690	0.009	1.285	0.087	0.896	0.025	1.111
	E+L+	0.003	1.642	0.013	1.205	0.113	0.844	0.033	1.056
Proventriculus	E-L-	0.090	0.561	0.070	0.627	0.057	0.636	0.074	0.601
	E-L+	0.079	0.596	0.077	0.601	0.073	0.611	0.077	0.602
	E+L-	0.182	0.373	0.085	0.576	0.029	0.765	0.074	0.596
	E+L+	0.184	0.362	0.087	0.569	0.036	0.781	0.074	0.543
Gizzard	E-L-	0.007	1.379	0.026	1.033	0.195	0.687	0.051	0.919
	E-L+	0.008	1.341	0.032	0.977	0.197	0.673	0.059	0.879
	E+L-	0.007	1.386	0.028	1.013	0.213	0.663	0.053	0.908
	E+L+	0.007	1.389	0.033	0.982	0.232	0.652	0.059	0.885
Pancreas	E-L-	0.001	1.241	0.002	1.137	0.003	1.072	0.002	1.122
	E-L+	0.001	1.268	0.002	1.128	0.004	1.000	0.003	1.069
	E+L-	0.001	1.324	0.002	1.165	0.004	1.009	0.002	1.104
	E+L+	0.002	1.153	0.002	1.084	0.003	1.041	0.003	1.078
Duodenum	E-L-	0.004	1.321	0.014	1.013	0.057	0.765	0.020	0.949
	E-L+	0.005	1.281	0.012	1.022	0.050	0.785	0.020	0.941
	E+L-	0.004	1.314	0.009	1.099	0.028	0.894	0.014	1.021
	E+L+	0.006	1.196	0.012	1.037	0.026	0.904	0.015	0.996
Jejunum	E-L-	0.003	1.488	0.014	1.112	0.084	0.809	0.027	1.007
	E-L+	0.004	1.482	0.016	1.081	0.127	0.731	0.035	0.951
	E+L-	0.003	1.488	0.014	1.108	0.095	0.779	0.028	0.994
	E+L+	0.005	1.405	0.018	1.054	0.103	0.757	0.032	0.955
Ileum	E-L-	0.010	1.196	0.025	0.974	0.077	0.779	0.033	0.927
	E-L+	0.013	1.142	0.026	0.943	0.077	0.762	0.037	0.888
	E+L-	0.011	1.171	0.026	0.943	0.086	0.738	0.036	0.888
	E+L+	0.022	0.990	0.031	0.900	0.051	0.818	0.035	0.874
Ceca	E-L-	0.000	1.706	0.002	1.178	0.021	0.788	0.005	1.054
	E-L+	0.000	1.766	0.001	1.263	0.024	0.746	0.005	1.038
	E+L-	0.000	1.797	0.001	1.235	0.014	0.804	0.004	1.041
	E+L+	0.000	1.716	0.002	1.185	0.018	0.815	0.004	1.053

E- = Low dietary AME; E+ = High dietary AME; L- = Low digestible Lysine; L+ = High digestible Lysine.

1 Coefficients for the allometric equation y=ax^b^; y=supply organ weight (g), x=yolk-free body weight (g); a and b estimated coefficients

**Table 12:** Results comparing the separate and pooled fits for each treatment combination of the allometric growth curves.

		D 0 and 4			D 0 and 11			D 4 and 11			D 0 and 4 and 11		
		E-L-	E-L+	E+L-	E-L-	E-L+	E+L-	E-L-	E-L+	E+L-	E-L-	E-L+	E+L-
Heart	E-L+	0.960			0.547			0.311			0.398		
	E+L-	0.239	0.245		0.682	0.398		0.585	0.639		0.398	0.637	
	E+L+	0.052	0.051	0.575	0.031	0.052	0.046	0.024	0.047	0.033	0.036	0.034	0.037
Liver	E-L+	0.274			0.177			0.003			0.054		
	E+L-	<0.001	<0.001		<0.001	<0.001		0.058	<0.001		<0.001	<0.001	
	E+L+	<0.001	<0.001	0.293	<0.001	<0.001	0.058	0.016	0.075	0.024	0.064	0.051	<0.001
Proventriculus	E-L+	0.147			0.682			0.193			0.511		
	E+L-	<0.001	<0.001		0.636	0.574		<0.001	<0.001		0547	0.390	
	E+L+	<0.001	<0.001	0.213	0.445	0.631	0.641	<0.001	<0.001	0.186	0.682	0.748	0.436
Gizzard	E-L+	0.674			0.059			0.633			0.487		
	E+L-	0.562	0.889		0.354	0.397		0.503	0.743		0.566	0.387	
	E+L+	0.488	0.648	0.761	0.051	0.418	0.407	0.549	0.802	0.463	0.696	0.741	0.640
Pancreas	E-L+	0.109			0.114			<0.001			<0.001		
	E+L-	<0.001	<0.001		0.056	0.052		<0.001	0.179		0.106	<0.001	
	E+L+	<0.001	<0.001	<0.001	<0.001	<0.001	<0.001	0.048	0.005	0.006	<0.001	0.132	<0.001
Duodenum	E-L+	0.989			0.886			0.745			0.637		
	E+L-	0.770	0.757		<0.001	<0.001		<0.001	<0.001		<0.001	<0.001	
	E+L+	<0.001	<0.001	<0.001	0.062	0.097	<0.001	<0.001	<0.001		<0.001	<0.001	0.075
Jejunum	E-L+	0.324			0.623			<0.001			<0.001		
	E+L-	0.391	0.609		0.364	0.109		0.136	<0.001		0.108	<0.001	
	E+L+	<0.001	<0.001	<0.001	<0.001	0.049	<0.001	<0.001	0.165	0.058	<0.001	0.111	0.047
Ileum	E-L+	0.166			<0.001			0.163			<0.001		
	E+L-	0.169	0.171		<0.001	0.104		0.001	0.283		<0.001	0.106	
	E+L+	<0.001	<0.001	<0.001	<0.001	<0.001	<0.001	0.149	<0.001	<0.001	<0.001	<0.001	<0.001

E- = Low dietary AME; E+ = High dietary AME; L- = Low digestible Lysine; L+ = High digestible Lysine.

^1^*P*-value results based on overall sum of squares fits using: F=((SS_pool_-(SS_trt1_+SS_trt2_))/(df_pool_-(df_trt1_+df_trt2_))/((SS_trt1_+SS_trt2_)-(df_trt1_+df_trt2_))

*P* < 0.05 indicates that the separate fit explains the variation better than the pooled fit, between the 2 treatment.

**Figure 2:**
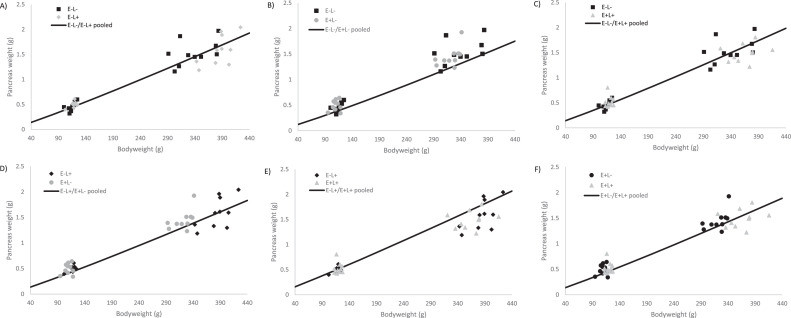
Treatment comparison of allometric growth curves of the pancreas using data from d 0, 4 and 11. The line shows the pooled allometric growth of 2 treatments, and the symbols represent measured liver weights. Graphs A, C, D, and F show that the separate fits explain the variation better, compared to the pooled fit. Whereas, graphs B and E show the separate fits and pooled fit both explain the variation.

The proventriculus displayed a higher allometric growth rate in the low AME groups from d 0 to 4, conversely from d 4 to 11 it was higher in the high AME groups. The allometric growth rate of the pancreas was higher in the E+L- group compared to the other groups from d 0 to 4, whereas it was lower in the E+L+ group during the same period. From d 4 to 11, the pancreas exhibited the highest allometric growth rate in the E-L- and the slowest in the E+L+, showing a reduction in diet density. However, from d 0 to 11, the pancreas had the lowest allometric growth rate in the E+L+ group compared to the others. When considering all data points, the allometric growth rate was higher in the high lysine groups (see Figure 2).

**Figure 3:**
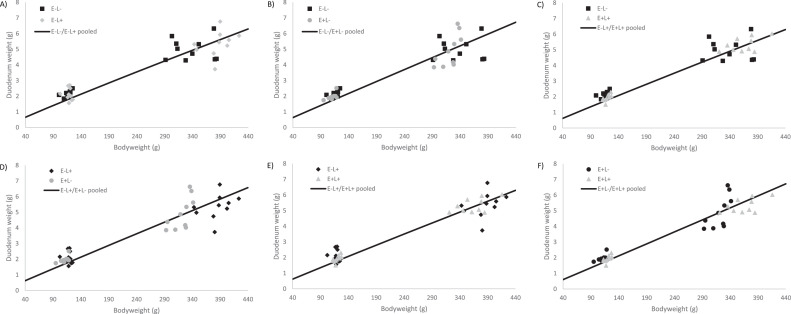
Treatment comparison of allometric growth curves of the duodenum using data from d 0, 4 and 11. The line shows the pooled allometric growth of 2 treatments, and the symbols represent measured liver weights. Graphs B, C, D, and E show that the separate fits explain the variation better, compared to the pooled fit. Whereas, graphs A and F show the separate fits and pooled fit both explain the variation.

**Figure 4:**
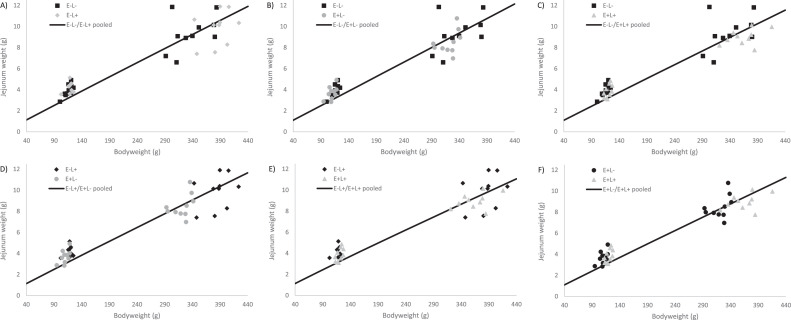
Treatment comparison of allometric growth curves of the duodenum using data from d 0, 4 and 11. The line shows the pooled allometric growth of 2 treatments, and the symbols represent measured liver weights. Graphs A, C, D, and F show that the separate fits explain the variation better, compared to the pooled fit. Whereas, graphs B and E show the separate fits and pooled fit both explain the variation.

Furthermore, the duodenal allometric growth was lower in the E+L+ group compared to the other groups from d 0 to 4, whereas from d 4 to 11, it was lower in the low AME groups. From d 0 to 11 the allometric growth was higher in E+L- compared to the other groups. When considering all data points, the allometric growth rate of the duodenum was higher in the high AME groups (see Figure 3). The jejunal allometric growth was lower in the E+L+ group compared to the other groups from d 0 to 4 and d 0 to 11. However, from d 4 to 11 and when considering all data points, allometric growth was higher in the high lysine groups (see Figure 4).

**Figure 5:**
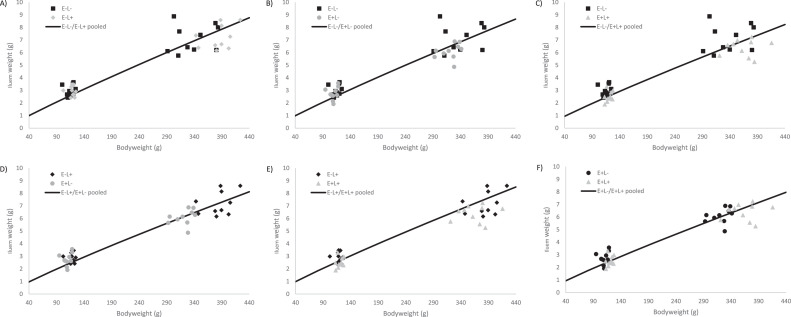
Treatment comparison of allometric growth curves of the ileum using data from d 0, 4 and 11. The line shows the pooled allometric growth of 2 treatments, and the symbols represent measured liver weights. Graphs A, B, C, E, and F show that the separate fits explain the variation better, compared to the pooled fit. Whereas, graph D shows the separate fits and pooled fit both explain the variation.

The allometric growth of the ileum was lower in the E+L+ group compared to the other groups from d 0 to 4. However, from d 4 to 11, allometric growth was higher in E+L+ compared to E-L+ and E+L-, and it was also higher in E-L- compared to E+L-. From d 0 to 11, and when considering all measurement points, allometric growth of the ileum was lower in the E-L- compared to the other groups, while E+L+ was lower compared to E-L+ and E+L- (see Figure 5). Notably, the other organs exhibited similar allometric growth across treatments.

## DISCUSSION

The purpose of the present study was to examine whether additional energy (from carbohydrates) or a higher amino acid density in the diet would support the (early) development of the supply organs.

### Growth Performance and Nutrient Intake

The observed decrease in BW gain in association with increasing AME from carbohydrates and reducing amino acid density underscores the intricate relationship between dietary composition and growth performance. Despite having similar grower and finisher feeds, the growth retardation observed in the starter phase was not compensated at a later age. With the addition of AME from carbohydrates no difference in BW gain from d 0 to 4 was observed; however, from d 4 to 11, it reduced BW gain. The lower BW gain from d 4 to 11 found in the current study as a result of lower amino acid density in the starter feed corresponds with earlier research (Swennen et al., 2010). Another consideration is the AME to Lys ratio, as deposition of body protein requires a balance of protein and energy. An lower amount of AME per mg of digestible lysine resulted in an increased BW gain from d 4 to 11 and d 11 to 28, this in contrast with the results of Plumstead et al., (2007) who reported no interaction between AME and Lysine in the first 21 d. However, FI plays an important role in these responses, based on evidence the broilers maintain an constant energy intake (Leeson et al., 1996).

Regulation of FI involves complex mechanisms influenced by nutrient signaling, and further complicating our understanding is the interplay with dietary composition, as feed intake is not the same as nutrient intake. Conflicting results from previous studies highlight the complexity of FI regulation. For instance, Swennen et al., 2010 reported that FI in the first week was unaffected by the starter feed composition; however, due to the focus on the isoenergetic substitution of one macronutrient, amino acid profile was not similar across treatments. Conversely, Noy and Sklan, 2002 reported a decreased FI with increasing dietary CP at stable digestible lysine level, resulting in lower body weight gain. The current study showed that higher AME combined with lower amino acid density reduced feed intake from d 0 to 4 and d 4 to 11, as well as d 11 to 28 in the common grower period. The other 2 studies appear to have different amino acid ratios in the starter diets across the treatments, which would be the most likely explanation for the differences seen, rather than dietary energy or lysine density.

The reduction in FI and subsequent nutrient intake can explain the lower BW gain in the low lysine groups in the current study. Findings from Lamot et al. (2019) suggested that an increased AME concentration through fat reduces FI based on the lipostatic theory. However, in the current study, AME was increased through carbohydrates. Applying the glucostatic theory, which suggests that hunger and satiety are primarily influenced by short-term shifts in blood glucose levels (King, 1976), could explain the lower FI with increased AME observed in the current study. However, the decreased glucoreceptor sensitivity in broiler chickens (Denbow et al., 1982), suggesting limitations in capacity to process carbohydrates is a more plausible explanation, potentially due to glycogen synthesis constraints. Additionally, an oversupply of starch combined with low amino acid levels can partially explain the reduced development observed, as an increase in starch availability has a negative impact on amino acid availability (Weurding et al., 2003; Moss et al., 2018). This suggests that a higher density of amino acids is needed to compensate for the increased amount of starch in feed, as seen in the E+L+ group. The impact of additional AME from carbohydrates at a young age appears to have a long-lasting negative effect on body weight.

### Supply Organ Weight, Histo-Morphological Parameters, and Allometric Growth

The relative weight of the heart showed no difference across treatments, however differences in developmental patterns were observed. Specifically, the growth rate of the heart was faster in the E+L+ group from d 4 to 11, as indicated by the higher parameter *b* estimations. A faster growth rate could suggest that the functional capacity of the heart is able to keep up with an overall higher body growth rate (Wideman et al., 2013). However, the current study showed that the growth rate of the heart had no effects on performance.

Relative liver weight was higher in the E+ groups and highest in the E+L- group on d 4, and also on d 11 the relative liver weight was still affected by the higher AME from carbohydrates. Overall, the growth rate (*b*) of the liver was above 1 in all treatment groups, indicating that the growth rate of the liver was faster than body growth, especially in the first days after hatching. The growth rate of the liver was faster in the E+ groups from d 0 to 4 and d 0 to 11. In the period from d 4 to 11 growth rate of the liver was faster in the L- groups; however, the *a* was lower in the period indicating the liver started with a lower weight on d 4. The fastest growth rate was found in the E+L- group, which matches the highest relative liver weight. This indicates that increased AME from carbohydrates with low amino acid density increases relative liver weight. Lamot et al. (2019) found that with increasing AME from fat combined with simultaneously increasing amino acid density the relative liver weight decreased. While amino acid density is the main difference between the study from Lamot and the current study it is unlikely that this explains the differences found in liver weight, given that no difference was observed in relative liver weight between the L- and L+ groups. Oxidation and storage of “excess” carbohydrates in the liver as glycogen can explain the increased relative weight found in this study, increasing the hepatic energy status (Scanes, 2022). To cope with the higher availability of carbohydrates, the catabolism of pyruvate in the liver is most likely increased, resulting in a higher pyruvate dehydrogenase activity to control blood glucose levels (Zhang et al., 2014). Higher pyruvate dehydrogenase has been shown to decrease feed intake (Roche et al., 2001), and this could explain the reduced feed intake seen in this study. This could suggest that within the glycolysis process itself, there is no rate-limiting step; however, it is the result of increased glycolysis, higher pyruvate dehydrogenase activity, that has a limiting effect on the amount of carbohydrates that can be consumed. This possibly explains why a high AME with low amino acid density leads to a higher growth rate of the liver, higher relative liver weight, and lower body weight gain.

Relative proventriculus weight was higher in the E- group at d 4, this corresponds with the higher growth rate in the E- group from d 0 to 4; whereas in the period from d 4 to 11 growth rate was slower in the E- group. This resulted in a similar growth rate across the treatment groups when data points from all d 0, 4, and 11 were included. The relative weight of the proventriculus was higher in the E-L- group, even with similar growth rates. (Hetland and Svihus, 2001) reported that a longer retention time of feed in the gizzard stimulated HCl secretion of the proventriculus via mechanoreceptors. An increased secretion of HCl could also lead to a relatively larger proventriculus and gizzard weight (González-Alvarado et al., 2008). The current study observed no difference in relative gizzard weight or different growth rate to explain the proventriculus weight through mechanical stimulation, and FI was also not reduced, which could indicate a slower passage rate. Particle size has also been linked to increased weight of the proventriculus (Jiménez-Moreno et al., 2010); but, although it was not measured, it is unlikely that particle size between the diets was markedly different. It could be that the lower nutrient density stimulated the proventriculus to increase HCl secretion.

Contrary to the expectations based on its regulatory role in carbohydrate metabolism and enzyme secretion, no differences in relative weight of the pancreas were observed. However, the growth rate of the pancreas was slower in the E+L+ group from d 0 to 4 and d 0 to 11; whereas in the total period of d 0, 4, and 11 the growth rate was faster in the L+ groups. This indicates that higher amino acid densities increase the growth rate of the pancreas. This is in line with (Swatson et al., 2002), who reported that increasing the amino acid density in a balanced profile resulted in a higher relative weight of the pancreas. Nevertheless, the growth rate (*b*) was above 1 in all groups, indicating that the growth rate of the pancreas was faster than body growth. It was expected that higher carbohydrate intake would increase pancreatic weight; however, weight and growth rate were similar for E- and E+. An explanation can be limiting glucagon production, given the strict homeostasis in birds. This could have resulted in a higher blood glucose level due to high carbohydrate intake, saturating the hexokinase IV-like receptor for poultry (Borrebaek et al., 2007), and reducing glucagon levels while increasing the glucose metabolism. This may, in part, explain the higher relative weight of the liver and lower BW gain as well (Printz et al., 1993b).

The effect of the starter diets on the small intestine differed between its segments. While relative duodenal weight showed only numerical differences across treatments, the growth rate was different following the line of the relative weight. When including all d (0, 4, and 11) the growth rate was slower in E- groups, whereas duodenal villus length was lower in the E+ on d 4 and 11. An oversupply of energy in the form of carbohydrates could possibly reduce the demand for monosaccharide transporters, leading to a reduction in villus growth. However, (Li et al., 2020) reported that diet did not affect gene expression patterns of monosaccharide transporters but reported no difference in villus development. The contradictive effect of the growth rate of the duodenum and villus length could be due to a relatively low impact of villus length on duodenal weight. For the jejunum, relative weight and length no differences were observed across all treatments; however, the growth rate when including all d (0, 4, and 11) was slower in the L+ groups. This is reflected in the numerical differences in the relative weight of the jejunum.

The relative weight of the ileum at d 4 and 11 was lowest in the E+L+ group and highest in the E-L- group, suggesting that a low-density diet increases development. The growth rate, when including all d (0, 4, and 11), shows the same effect, the lowest *b* value is found in the E+L+ and the highest in the E-L- group. Some starch digestion, less than 10%, has been reported in the ileum (Svihus et al., 2004, Zimonja and Svihus, 2009); however, the development of the ileum is most likely more related to nutrient density than starch. The growth rate of the duodenum, jejunum, and ileum show different effects when including all d (0,4, and 11). In the period from d 0 to 4 growth rate for the entire small intestine is lower in the E+L- group. A possible explanation is that in this period the higher readily available starch in relation to lysine reduces growth rate of the small intestine (Herwig et al., 2020), resulting in a lower overall performance also at a later age.

It could be hypothesized that the observed variations in supply organ weights could be attributed not only to the lysine content but also to secondary differences in soybean meal and fiber resulting from the feed formulation process. Besides the crude protein, soybean meal contributes also other bioactive factors and anti-nutritional factors that may impacted the GI development at young age (Batal and Parsons, 2002). Furthermore, fiber may stimulate GIT development, while excessive fiber may also result in increased endogenous protein losses. However, further research is necessary to clarify these potential effects.

Despite several studies demonstrating differences in goblet cell populations with age or dietary changes (Langhout et al., 1999; Liu et al., 2020; Reynolds et al., 2020; Uni et al., 2003a), the current study observed no effect on the goblet cell population, suggesting that it is not likely that total increased amino acid density or additional energy from carbohydrates affect goblet cell development.

In conclusion, feeding a starter diet with high AME from carbohydrates with low amino acid density has a long-lasting negative effect on performance. Both high AME from carbohydrates and low amino acid density appear to change the growth pattern of the supply organs, increasing relative liver weight and growth rate, lowering duodenal villus length, and slowing the growth rate of the ileum, combined with no response observed on weight or growth rate of the pancreas. The effects of high AME from carbohydrates and low amino acid density on the supply organs ultimately resulted in lower BW gain and FI in the starter period, which subsequently reduces feed intake and lower BW gain from d 0 to 35. The detrimental effects of an increased AME from carbohydrates in feed can be partially compensated by increasing the amino acid density in the diet.

## DISCLOSURES

The authors declare no conflicts of interest.
